# 8-Oxoguanine Affects DNA Backbone Conformation in the *Eco*RI Recognition Site and Inhibits Its Cleavage by the Enzyme

**DOI:** 10.1371/journal.pone.0164424

**Published:** 2016-10-17

**Authors:** Joanna J. Hoppins, David R. Gruber, Heather L. Miears, Alexey S. Kiryutin, Rustem D. Kasymov, Darya V. Petrova, Anton V. Endutkin, Alexander V. Popov, Alexandra V. Yurkovskaya, Stanislav O. Fedechkin, Jacob A. Brockerman, Dmitry O. Zharkov, Serge L. Smirnov

**Affiliations:** 1 Chemistry Department, Western Washington University, Bellingham, WA, United States of America; 2 SB RAS International Tomography Center, Novosibirsk, Russia; 3 Novosibirsk State University, Novosibirsk, Russia; 4 SB RAS Institute of Chemical Biology and Fundamental Medicine, Novosibirsk, Russia; 5 Institute of Biochemistry, Stuttgart University, Stuttgart, Germany; 6 University of California Santa Cruz, Program in Biomedical Science and Engineering, Santa Cruz, CA, United States of America; 7 Department of Biochemistry and Molecular Biology, University of British Columbia, Vancouver, BC, Canada; New England Biolabs Inc, UNITED STATES

## Abstract

8-oxoguanine is one of the most abundant and impactful oxidative DNA lesions. However, the reasons underlying its effects, especially those not directly explained by the altered base pairing ability, are poorly understood. We report the effect of the lesion on the action of *Eco*RI, a widely used restriction endonuclease. Introduction of 8-oxoguanine inside, or adjacent to, the GAATTC recognition site embedded within the Drew—Dickerson dodecamer sequence notably reduced the *Eco*RI activity. Solution NMR revealed that 8-oxoguanine in the DNA duplex causes substantial alterations in the sugar—phosphate backbone conformation, inducing a BI→BII transition. Moreover, molecular dynamics of the complex suggested that 8-oxoguanine, although does not disrupt the sequence-specific contacts formed by the enzyme with DNA, shifts the distribution of BI/BII backbone conformers. Based on our data, we propose that the disruption of enzymatic cleavage can be linked with the altered backbone conformation and dynamics in the free oxidized DNA substrate and, possibly, at the protein—DNA interface.

## Introduction

8-oxoguanine (oxoG) is a naturally abundant base lesion with a well characterized mutagenic potential [1]. It is easily generated in DNA by reactive oxygen species endogenously produced during normal aerobic metabolism as well as by environmental assaults such as ionizing radiation. Under normal circumstances, the persisting background level of oxoG in human DNA has been estimated as ~0.5–5 oxoG / 10^6^ guanines [2]; however, oxidative stress and many diseases cause a significant increase in the amount of this lesion [3–5]. Due to its propensity to pair with adenine during replication, oxoG induces G:C→T:A transversions, which are currently regarded as the lesion’s main biological consequence [1,6].

Aside from its miscoding properties, oxoG has been shown to affect recognition of DNA by some DNA-binding proteins. The presence of oxoG in the recognition sequence has been shown to inhibit DNA cleavage by several restriction endonucleases, including *Hae*III, *Hpa*II, *Msp*I, *Nhe*I and *Ngo*MIV [7–10]. Oxidation of G in CpG dinucleotide substantially alters methylation of the C within the site by human DNA methyltransferases DNMT1 and DNMT3a [11,12]. The presence of oxoG in the topoisomerase I recognition site, depending on the position, may either enhance or suppress the enzyme activity [13,14]. Transcription factors AP-1 and Sp1, methyl-CpG-binding protein MeCP2, and telomere protecting proteins TERF1 and TERF2 poorly bind oxoG-containing recognition sequences [15–17], whereas binding of NF-κB to its promoter element may either decrease or increase depending on oxoG location in the sequence [18]. This interference of oxoG with normal protein—DNA interactions may potentially have biological consequences such as improper regulation of gene expression, continuing survival of the invasive foreign DNA with a damaged restriction site in a bacterial cell, etc. [19,20].

The exact reasons of the differences observed in enzymatic recognition of the DNA substrate upon introduction of the oxoG remain unclear, especially when the lesion does not form direct contacts with the protein. To explain specific changes in protein—DNA recognition, a comprehensive analysis of DNA conformation is required, including within the backbone and the base pairs [21]. Within the same helix geometry, backbone dihedral angles define different types of DNA backbone conformation (e.g. BI *vs* BII) [22]. In this paper, we have used restriction endonuclease *Eco*RI as a model to analyze possible effect of oxoG in protein—DNA interactions. *Eco*RI was selected for the following reasons: (i) this is a well-studied enzyme, with the activity towards many other deviations from the cognate site described experimentally [23–25]; (ii) oxoG affects the recognition of DNA by several other structurally unrelated restriction enzymes (see above); (iii) the structure of *Eco*RI bound to its cognate DNA is available [26], allowing the analysis of direct protein—DNA interactions; (iv) the *Eco*RI site -GAATTC- is a central part of extensively characterized Drew—Dickerson dodecamer [27,28], which provides an excellent reference point for comparison of the undamaged and damaged cognate site in free DNA. We report that the activity of restriction endonuclease *Eco*RI is inhibited by oxoGplaced within, or adjacent to, the recognition site despite the apparent conservation of the protein—DNA interface. Our solution NMR analysis of oxoG-containing DNA substrates provides a possible structural explanation (backbone alteration) of this effect.

## Materials and Methods

### Oligonucleotides and enzymes

Sequences of the oligonucleotides used in this study are listed in Table 1. Oligonucleotides were synthesized in-house from commercially available phosphoramidites (Glen Research, Sterling, VA). Restriction endonuclease *Eco*RI was from SibEnzyme (Novosibirsk, Russia). *E*. *coli* Fpg was purified as described [29].

**Table 1:** Sequences of oligonucleotides used in this study.

ID	Sequence*
**DDD**	**CGCGAATTCGCG**
oxoG4	**CGC****X****AATTCGCG**
oxoG10	**CGCGAATTC****X****CG**
8-10-G	CCTGCTTT**CGCGAATTCGCG**TCCACGGCAC
8-10-X1	CCTGCTTT**CGC****X****AATTCGCG**TCCACGGCAC
8-10-X2	CCTGCTTT**CGCGAATTC****X****CG**TCCACGGCAC
10-8-G	GTGCCGTGGA**CGCGAATTCGCG**AAAGCAGG
10-8-X1	GTGCCGTGGA**CGC****X****AATTCGCG**AAAGCAGG
10-8-X2	GTGCCGTGGA**CGCGAATTC****X****CG**AAAGCAGG

*X = 8-oxoguanine;

**DDD** (Drew—Dickerson dodecamer, common for all the sequences).

### Enzyme activity and kinetics

The reaction mixture included 100 mM Tris-HCl (pH 7.6), 50 mM NaCl, 10 mM MgCl_2_, 1 mM dithiothreitol, 1.5–100 nM ^32^P-labeled oligonucleotide duplex, and 0.4 or 2 U/μl *Eco*RI (approximately 60 or 300 pM, respectively, for kinetics and single-point assay). The reaction was allowed to proceed for 20 min, terminated by adding an equal amount of formamide/EDTA and heating for 5 min at 95°C, and analyzed by electrophoresis in 20% polyacrylamide gel with 8 M urea. The gel was quantified using Molecular Imager FX with Quantity One v3.1 software (Bio-Rad, Hercules, CA).

### NMR experiments

NMR data were collected utilizing 700 Bruker NMR spectrometer (^1^H 700 MHz) equipped with a Prodigy TCI cryoprobe and a BBO probe for the ^31^P-^1^H correlation spectra. For the NMR recordings, the duplex DNA samples were dissolved in the following buffer: 10 mM Na phosphate (pH 6.8), 50 mM NaCl, 1 mM EDTA.

The NMR data were processed with NMRPipe software [30] and the spectra were visualized with NMRDraw [30] and NMRView [31]. The NMR resonance assignment was performed by the standard procedure [28] utilizing the sets of 2D NOESY (70, 140 and 200 ms mixing time, 100% ^2^H_2_O buffer), the 2D TOCSY (100% ^2^H_2_O buffer) as well as the 2D NOESY (90% H_2_O buffer) data. The assignment of ^31^P NMR resonances was performed utilizing the standard protocol [32].

The distance restraints were derived from the NOESY cross-peaks generated with NMRView. The volume-to-distance calibration of the NOESY peaks’ volumes was performed using the CYANA 2.1 software [33] with cytosine H5–H6 distance as the calibration standard. When reliably available from all the NOESY spectra collected, every final NOE restraint was calculated as their “averaged” distance. In all other cases, every final NOE restraint was derived from their corresponding most reliable NOESY cross-peak (“non-averaged”). All the NOE restraints were duplicated for the other (self-complimentary) strand for both samples. The obtained distance restraints were grouped according to their length: short (< 3.0 Å), medium (between 3.0 and 5.0 Å) and long (> 5.0 Å) distances as well as methyl-containing restraints. The group-specific no-penalty tolerances were employed based on the NOE distances and spectral quality (S1 Table). The standard Watson—Crick hydrogen bonding distance restraints were applied for all the base pairs based on the NOESY cross-peak patterns observed in 90% H_2_O. No sugar conformation restraints were used. Based on the observed ^31^P resonances, the backbone dihedrals were restrained to a standard BI conformation [34] for all the canonical residues, whereas the oxoG nucleotide had the backbone angle ε restrained within 215–295° and ζ within 155–205° range (BII conformation). The dihedral angles adjacent to ε and ζ (two on either side) were allowed to sample a broader range of values (by ± 20°).

### Restrained molecular dynamics

Restrained molecular dynamics (rMD) simulations were conducted via a simulated annealing protocol with AMBER 12 [35] using the parm99 force field [36]. The generalized Born (GB) implicit solvation model [37] was used. The starting structures used were of A- and B- DNA type models. The oxoG-specific force field parameters, including the electric charges, were taken from [38]. The protocol for energy minimization, restraints and simulated annealing is included in S1 Document. A total of 18 structures were produced for each sample. The ensemble of structures was determined based on the lowest conformational penalty/energy values. A representative structure for each ensemble was built by averaging the lowest energy models and subjecting the obtained frames to energy minimization.

### Free molecular dynamics

Free molecular dynamics (fMD) simulations were performed using PMEMD from AMBER 12. Each structure was parameterized with the AMBER ff99SB force field [39] with parmbsc0 modifications specific to the DNA backbone [40]. Additional information regarding initialization, minimization and equilibration protocols is included in S1 Document. Production molecular dynamics simulations were run for 120 ns using GPU code with the PMEMD.cuda implementation of SANDER [41] from AMBER 12. Long range interactions were calculated with PME. A 2 fs integration time step was used. Simulation coordinates were recorded every 1 ps. The first 10 ns of each simulation were removed giving 110 ns total simulation time for each sample to analyze.

### Hybrid explicit/implicit solvent molecular dynamics

The starting model for the MD analysis of *Eco*RI bound to oxoG-containing DNA was the X-ray structure of pre-transition state *Eco*RI in a complex with a 13-mer duplex containing the GAATCC sequence (PDB ID 1CKQ). Both G bases in the recognition sequence were changed to oxoG. Heavy atoms missing in the structure and hydrogen atoms were restored using the Missing Atom Restoration module of BioPASED molecular modeling package [42]. Out of 302 water molecules found in the crystal unit cell, 129 tightly bound molecules were identified using the WaterShell optimization algorithm [43] and retained as explicit water, otherwise the modeling was done in EEF1 implicit solvent [44] with counter-ion correction charge scaling [45] to enhance the sampled conformational space [46]. All models were energy-minimized in 500 steps of Fletcher energy optimization followed by 100 ps of simulated annealing using the BioPASED package. Molecular dynamics simulations (12 ns) were performed using BioPASED with the AMBER ff99SB force field [39] with parmbsc0 correction [40]; the integration time step was 1 fs. The system was gradually heated from 10 K to 300 K in 50 ps and equilibrated at this temperature. Classic NVT ensemble molecular dynamics trajectories were generated with harmonic restraints of 0.25 kcal/Å^2^ for the terminal nucleotides and 0.001 kcal/Å^2^ for the rest of heavy atoms except amino acid side chains that were modeled with no restraints. Simulation coordinates were recorded every 2 ps.

### Structure visualization and analysis

The structures were visualized and analyzed with VMD [47] and Chimera [48]. The molecular dynamics trajectories were analyzed using CPPTRAJ [49], Curves+ [50], 3DNA [51], MDTRA [52] and custom Python scripts.

## Results

### Effect of oxoG on the GAATTC sequence recognition by EcoRI

In an attempt to follow possible biological consequences of oxoG in DNA, we have compared the activity of *Eco*RI restriction endonuclease on its natural substrate and in several contexts containing oxoG. A 30-mer duplex contained a palindromic 12-mer CGC**G**AATTC**G**CG sequence (Drew—Dickerson dodecamer, DDD) in the middle flanked by non-palindromic shoulders. One of the guanines in the recognition site GAATTC, or a guanine immediately 3′ to this site, was replaced with oxoG (bold and underlined in the sequence above). The cleavage of either the oxidized strand or the opposite strand was followed.

**Fig 1:**
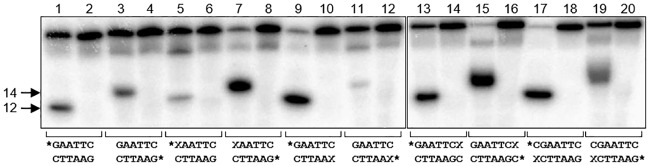
Cleavage of native and oxoG-modified GAATTC sequence by *Eco*RI. Odd lanes, enzyme added (300 pM); even lanes, no enzyme (controls). The concentration of the substrate was 100 nM. The central part of the 30-mer duplexes and the position of oxoG (X) and radioactive label (asterisk) are shown below the image. Cleavage of the top strand produces a 12-mer product; cleavage of the bottom strand produces a 14-mer product.

In the crystal structure of *Eco*RI, the guanine base in the recognition sequence forms no direct hydrogen bond with the protein [26], yet its replacement with isosteric bases 7-deazaguanine or 2-aminopurine leads to ~60-fold drop in the enzyme activity [53]. In agreement with these observations, introduction of oxoG in the recognition sequence severely suppressed the cleavage of the modified strand (Fig 1, Table 2). The steady-state kinetic parameters of cleavage of the non-damaged strand were compromised, but the reaction still proceeded to completion with more enzyme (Fig 1, Table 2). In an unrelated 18-mer duplex containing the GAATTC site not embedded within the Drew—Dickerson context, the introduction of oxoG inhibited the cleavage almost completely in the modified strand and reduced it in the opposite strand (S1 Fig). When oxoG was introduced next to the GAATTC site, as in the Drew—Dickerson sequence, it increased *K*_M_ and decreased *V*_max_ of nicking of the modified strand, although the reaction was also efficient with the larger amounts of the enzyme (Fig 1, Table 2). Since *Eco*RI cleavage is rate-limited by the release of doubly nicked product [54], the apparent increase in the efficiency of cleavage with the concomitant decrease in the *V*_max_/*K*_M_ ratio may be due to the change in the rate-limiting step when oxoG is present in the substrate. Regardless of the kinetic scheme, it is clear that oxoG affects the recognition or cleavage of the cognate site by *Eco*RI.

**Table 2:** Kinetics of EcoRI on oxoG-containing substrates.

Sequence of the *Eco*RI site	*K*_M_, nM	*V*_max_, nM·min^–1^	*V*_max_/*K*_M_, min^–1^	relative to undamaged^#^
*-**GAATTC**G- -**CTTAAG**C-	20.0 ± 5.0	0.30 ± 0.03	(15 ± 4)×10^−3^	1
*-**XAATTCG**- -**CTTAAG**C-	n/d*			
*-**GAATTC**G- -**CTTAAX**C-	n/d*	n/d*	(0.31 ± 0.04)×10^−3^	0.021
*-**GAATTC**X- -**CTTAAG**C-	40.0 ± 13.0	0.067 ± 0.010	(1.7 ± 0.6)×10^−3^	0.11

*n/d, saturation by the substrate could not be achieved; *K*_M_ and *V*_max_ were not determined individually; *V*_max_/*K*_M_, when possible, was determined from the linear slope of the *v*_0_ vs [S] graph.

^#^Based on the *V*_max_/*K*_M_ ratio.

### OxoG in the recognition sequence does not affect the structure of EcoRI—DNA complex

**Fig 2:**
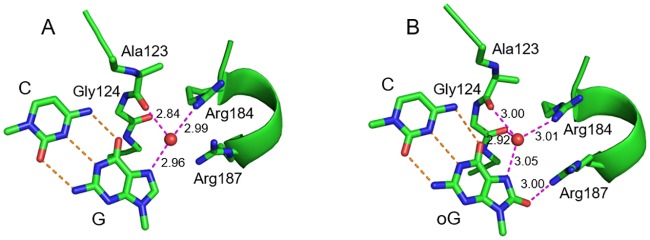
Structures of the G:C pair in the *Eco*RI recognition site from molecular dynamic simulation. The representative snapshots (10 ns) for native (A) and oxoG-modified (B) cognate site are shown. Carbon atoms and secondary structure elements are colored green, oxygens, red, nitrogens, blue. For DNA, only the G:C or oxoG:C base pair is shown. Orange dashed lines indicate Watson—Crick bonds, magenta dashed lines show hydrogen bonds with the protein formed by G or oxoG directly or through the interface water molecule (red ball). Lengths of hydrogen bonds (Å) are shown next to the lines.

In the crystal structure of pre-transition state *Eco*RI—DNA complex (PDB 1CKQ), the N7 atom of the guanine base in the recognition sequence accepts an H-bonding interaction with a water molecule that, in turn, is coordinated by the carbonyl oxygen of Ala123 and Nη1 of Arg184 (Fig 2A). Since N7 in oxoG is a hydrogen bond donor rather than acceptor, we performed molecular dynamics of *Eco*RI bound to either native or oxoG-modified GAATTC site to assess how this change might affect the structure of the enzyme—DNA interface. No significant deviations in the occurrence or energy of bonds existing between an *Eco*RI dimer and the recognition site, or within base pairs in the recognition site were observed (S2 Table). The N7-bound water molecule simply re-oriented to accept a hydrogen bond from N7 of oxoG, while keeping the HOH···O[Ala123] and Nη1[Arg184]···HOH bonds (Fig 2B). Moreover, in the oxoG-modified structure, the water molecule additionally donated a hydrogen bond to the carbonyl oxygen of Gly124, and O^8^ of oxoG accepted a hydrogen bond from Nη1 of Arg187 (Fig 2B). We conclude that loss of activity of *Eco*RI on oxoG-modified recognition site is unlikely to stem from the local perturbation of the enzyme—DNA interface in the pre-incision complex by the lesion. Notably, while all non-terminal nucleotides in the G model were in BI conformation, the oxoG model spontaneously produced a significant fraction of BII in the C nucleotide opposite the lesion (S2 Fig). This backbone effect was the only significant difference observed between DNA in G and oxoG models.

### NMR characterization of normal and damaged duplexes

In an attempt to uncover possible reasons for *Eco*RI inhibition by oxoG, we have determined solution structures of two duplex DNA samples derived from the Drew—Dickerson dodecamer sequence ([27], DDD): oxoG4 and oxoG10 (Table 1). The oxoG4 sample represents guanine oxidation within the *Eco*RI recognition site, whereas the oxoG10 sequence has the oxoG lesion flanking this site. In the context of our enzyme kinetics study, oxoG4 corresponds to the 8-10-X1 and 10-8-X1 oligonucleotides, whereas oxoG10 corresponds to the 8-10-X2 and 10-8-X2 counterparts (Table 1).

The composition of the peaks on all the 2D NMR spectra for oxoG4 indicates that the sample adopts a single distinct A/B-DNA conformation. The same observation was made for oxoG10. The NMR resonance assignment for oxoG4 and oxoG10 duplexes was performed by the previously reported procedure [55] utilizing the following 2D NMR data: NOESY (100% ^2^H_2_O buffer); TOCSY (100% ^2^H_2_O buffer); and NOESY (90% H_2_O buffer). The 2D NOESY peaks patterns for both samples are highly similar to those reported in the unmodified sequence, a *de facto* A/B DNA standard [28]. The main differences are localized around the oxoG sites (S3 and S4 Figs). The 2D NOESY spectra of the oxoG4 sample recorded in 100% ^2^H_2_O yielded a total of 189 unique NOEs, with 82 of them being “averaged”, 94 “non-averaged” and 13 methyl-containing restraints. The oxoG10 structure was driven by 233 unique NOEs with 120 “averaged”, 90 “non-averaged” and 23 methyl-containing restraints. The DQF-COSY H1′···H2′/H2″ cross-peak patterns, whenever unobscured, established that the sugar conformations of all the nucleotides were in the C2′-*endo*/C3′-*exo* range. The 2D NOESY recorded in 90% H_2_O shows the distinct set of cross-peaks linking protons from the bases on the opposite strands and confirming the Watson—Crick pattern of H-bonding within the non-terminal base pairs (S4 Fig). NMR spectral features involving oxoG protons were as expected (BMRB IDs 25369 and 25528): absence of purine H8 NOESY cross-peaks (S3 Fig), downfield shift of H2″ (> 2.33 ppm), and presence of peaks involving HN7 in spectra recorded in the 90% H_2_O buffer.

Both 1D and 2D ^31^P NMR spectra for the two samples show the majority of ^31^P resonances clustered within 0.5 ppm as previously reported for the unmodified sequence [56] and a single peak from oxoG phosphate group, shifted downfield by ~0.3 ppm (S5 Fig). The chemical shift value of the oxoG ^31^P resonance indicates a more rare BII backbone conformation [22] unlike the common BI conformation of all other nucleotides.

### Solution NMR structure of oxoG-containing duplexes

**Fig 3:**
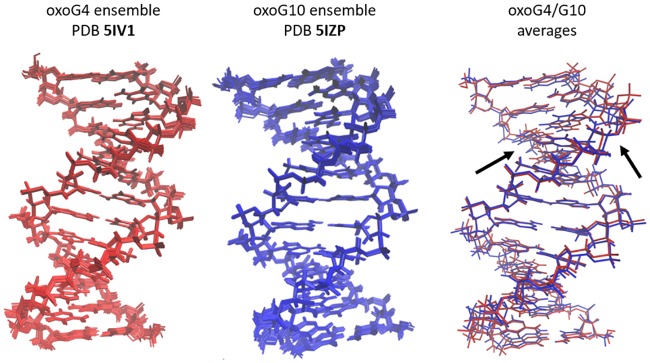
Ensembles and representative structures for oxoG4 and oxoG10. The entirely red ensemble (panel on the left) shows the 13 lowest energy structures and the averaged minimized structure for oxoG4, PDB ID 5IV1. The entirely blue ensemble (middle panel) shows the 14 lowest energy structures and the averaged minimized structure for oxoG10, PDB ID 5IZP. The panel on the right shows an overlay of the averaged minimized structures for both oxoG4 and oxoG10, in red and blue, respectively. In this last panel, the arrows show the general location of the two modified base pairs, C_3_:oxoG_4_ and C_9_:oxoG_10_.

An ensemble of lowest-energy structures was derived from rMD simulations: 13 models for oxoG4 and 14 for oxoG10 (Fig 3). The greatest NOE restraint violation for any model was 0.10 Å and dihedral violations did not exceed 11°. Within each ensemble, the largest pairwise all-atom RMSD value was less than 0.92 Å. The main structural differences between samples oxoG4 and oxoG10 were localized at their respective modification sites (Fig 3).

**Fig 4:**
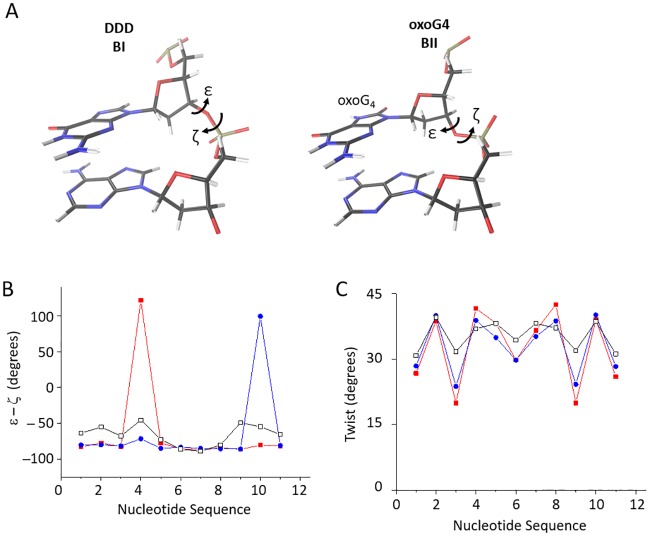
Results from the NMR-driven restrained molecular dynamics simulation of oxoG4 and oxoG10. (A) The difference in backbone conformation between BI and BII conformations. ε and ζ are highlighted in accordance to their torsion angle definition, as BI and BII are defined by the ε–ζ. For both DDD BI and oxoG4 BII structures, the G_4_ and A_5_ are shown and rotated to highlight the backbone differences. The DDD BI conformation (left panel) is from the PDB ID INAJ structure. The BII conformation induced by oxoG4 (right panel) is from our averaged minimized structure, PDB ID 5IV1. Carbons are shown in black, oxygen is shown in red, nitrogen in blue and hydrogens in white. The additional oxygen at C8 and hydrogen at N7 in the oxoG_4_ base are color-coded in the same way. (B) The ε–ζ shows BII directly 3' of the modification site for oxoG4 and oxoG10. BI is defined as ε–ζ less than 20°, with over 20° defined as BII. The nucleotide sequence numbers correspond to the steps in the sequence, with ε–ζ for nucleotide step 1 corresponding to the torsion angles between C_1_ and G_2_ in C_1_G_2_C_3_G_4_A_5_A_6_T_7_T_8_C_9_G_10_C_11_G_12_. In black with open square markers, are the ε–ζ for 1NAJ (DDD). In red with closed square markers are ε–ζ for oxoG4 and blue with closed circle markers are oxoG10. (C) oxoG4 and oxoG10 cause significant unwinding near the modification site. Color schemes and data origins are the same as in panel B.

Overall, the structures of oxoG4 and oxoG10 are very similar to each other (backbone RMSD of 1.2 Å between the representative oxoG4 and oxoG10 structures, Fig 3) as well as to known DDD structures (PDB 1BNA, 355D and 1NAJ; backbone RMSD of 0.9–1.4 Å). The major structural perturbation of our oxidized samples with respect to B-DNA is the switch of the backbone to BII conformation 3' to oxoG (Fig 4A and 4B). In addition, the *twist* helical parameter adopts unusually low values for the base-pairs adjacent to oxoG nucleotides from the 5′ side (Fig 4C). This unwinding effect was previously predicted based on the free molecular dynamics modeling [57] and is also demonstrated in our free MD analysis below.

### Free molecular dynamics of oxoG4 and oxoG10

The coordinates of the last 110 ns of the free dynamics were used to build a representative, averaged structure for each sample. The structures of oxoG4 and oxoG10 are very similar to each other (backbone RMSD of 0.89 Å) as well as to the unmodified DDD (backbone RMSD of 1.4 Å with respect to 1NAJ). The base pair parameters and backbone torsion angles of these average structures were similar to those of our solution NMR structures. Any significant conformational differences were observed only at or around the oxoG sites (S6 and S7 Figs). Introduction of oxoG causes a decrease in *twist* value for the oxoG-containing CpG base pairs (S6 Fig). The simulations also yielded a decrease in the ζ backbone torsion angle and an increase in the ε angle values for the oxoGpA and oxoGpC steps (in oxoG4 and oxoG10 respectively). As a result, the backbone adopts the BII conformation 3′ to oxoG as opposed to the BI state seen elsewhere in the duplex (S7 Fig).

## Discussion

8-Oxoguanine is a pro-mutagenic base constantly generated in DNA and removed through base excision repair. Chemically, it deviates from guanine only by two positions, which do not affect the Watson—Crick edge: oxygen replaces hydrogen at C8, and N7 is protonated. However, the effect of these changes is propagated to other physicochemical properties, some of which are used by DNA repair enzymes to facilitate oxoG recognition. Compared with G, oxoG thermodynamically destabilizes DNA [58], carries a differently oriented dipole [59], has p*K*_a_ of N1 lower by ~1 pH unit [60], and is more easily oxidized [61]. Moreover, oxoG can perturb the DNA structure. One example is oxoG:A mispair where oxoG adopts a *syn* conformation possibly due to steric repulsion involving O^8^ [55]. The BII conformation of the backbone 3' of oxoG in Watson—Crick base pairs is another major oxoG-related structural alteration, which was predicted computationally [57], present (but not described) in a crystal DNA structure [62] and is shown for the first time in solution NMR structures in this work.

The presence of oxoG in DNA interferes with its cleavage by several restriction endonucleases [7–10] and binding by several regulatory proteins [15–18]. Our work shows that oxidation of guanine within the *Eco*RI recognition site suppresses the endonuclease activity on the modified strand (Fig 1, S1 Fig). Although in some cases, such as *Msp*I and *Ngo*MIV endonucleases [63,64] or AP-1 Jun—Fos heterodimer [65], this effect can be directly attributed to the loss of interactions at the protein—DNA interface, *Eco*RI seems to conserve the full set of interactions with oxoG-containing recognition sequence as indicated by our MD simulations (Fig 2). Based on our structure and function data, we suggest that conformational changes effected by oxoG on DNA, particularly on the sugar—phosphate backbone, may be responsible for this reduction in *Eco*RI activity. Our solution NMR spectra and structures as well as free Molecular Dynamics data show that oxoG causes dramatic unwinding of the DNA helix 5′ to oxoG and pushes the backbone into the BII conformation 3′ to oxoG (Fig 4A and 4B and S5 and S7 Figs). The local unwinding (lower twist, Fig 4C, S6 Fig) could hinder either the recognition step or the following DNA kinking necessary for *Eco*RI activity. Both phosphates flanking G_1_ (G_4_ in our DDD sequence) in the *Eco*RI recognition sequence (G_1_A_2_A_3_T_4_T_5_C_6_) are part of the “primary clamp”, the set of phosphates identified by interference experiments that are required for proper formation of the catalytically competent complex [53] [66]. A similar observation of backbone conformation affecting *Eco*RI activity was made for a series of 2′-substituted G1 nucleotides, where the *gauche-gauche* to *gauche-trans* shift in the GpA phosphodiester bond rendered it non-scissile [67]. Therefore, we suggest that the oxoG lesion could affect backbone-mediated and indirect DNA recognition by other sequence-dependent proteins.

## Supporting Information

S1 Document Parameters and procedures for the restrained and free molecular dynamics simulation (rMD and fMD respectively). (PDF)

S1 Fig The oxoG modification in the GAATTC site inhibits its cleavage by *Eco*RI in a sequence context different from the Drew—Dickerson dodecamer. Odd lanes, no enzyme; even lanes, *Eco*RI. The asterisk marks the ^32^P-labeled chain. X = oxoG. The sequence of the *Eco*RI site-bearing duplex is shown.
(TIF)

S2 Fig The oxoG modification causes the BI/BII equilibrium shift in the cognate GAATTC site bound to *Eco*RI as observed in the molecular dynamics of the complex. (TIF)

S3 Fig 2D NOESY spectra (in 100% ^2^H_2_O) for oxoG4 and oxoG10 highlighting the “walk” region. The dodecamer sequence was confirmed to be symmetric, right handed helix within this region. The cross-peaks between the base proton H6/H8 and the H1’ of current or next bases (the “walk”) lead to the NMR resonance assignment (base protons labeled). The number of peaks shown confirms the loss of the guanine H8 at the appropriate modification site for both oxoG4 and oxoG10.
(TIF)

S4 Fig The spectra recorded in 90% H_2_O for oxoG4 and oxoG10 highlighting the imino region. (A) 2D NOESY spectra highlighting the Watson-Crick base pairing interactions in oxoG4 and oxoG10. Three separate CG base pairs are observed for both samples, with the resonances from terminal base pairs not showing due to their decreased thermal stability. Two distinct AT base pairs are also observed for both samples. (B) The imino proton regions of the 1D spectra. The modified residues contain an additional hydrogen at N7 (HN7) that is not seen in canonical guanines. The peaks marked with arrows correspond to the HN7 in both samples.
(TIF)

S5 Fig The 2D ^1^H-^31^P HETCOR spectra for oxoG4 and oxoG10 showing the interactions between the H3' of a residue and the P of the adjacent residue. Labels are provided to highlight the residue whose H3' corresponds to that specific peak, all phosphates belong to the residue on the 3' end of the labelled residue. In both spectra, a significant shift is seen in the ^31^P dimension of the modified residue, demonstrating evidence for that the backbone on the 3' end of the oxidized guanine residue is in BII, and all others are in the canonical BI form.
(TIF)

S6 Fig The oxoG modification causes significant unwinding around the modification site in oxoG4 and oxoG10 as observed in free molecular dynamics simulations. The nucleotide sequence numbers correspond to the steps in the sequence, with the twist for nucleotide step 1 corresponding to the twist of the first CG base pair with respect to the next one. In black with open square markers, are the DDD twist for the unmodified DDD. In red with closed square markers are the twist for oxoG4 and blue with closed circle markers are oxoG10.
(TIF)

S7 Fig The oxoG modification causes the BI frequency decrease (BII frequency increase) at the modification site as observed in the free molecular dynamics of oxoG4 and oxoG10 for one strand (the other strand’s data are highly similar). The nucleotide sequence numbers correspond to the steps in the sequence, with the twist for nucleotide step 1 corresponding to the backbone step between the C_1_ and G_2_ in DDD sequence. In black with open square markers, are the DDD twist for the unmodified DDD. In red with closed square markers are the twist for oxoG4 and blue with closed circle markers are oxoG10.
(TIF)

S1 Table Tolerances applied to NOE distance restraints in structure calculations. (PDF)

S2 Table Hydrogen bonds observed in the GAATTC site during molecular dynamics simulation of *Eco*RI in a complex with DNA containing G or oxoG at the 1^st^ position of the recognition sequence*. (PDF)

## Abbreviations

oxoG: 8-oxoguanine
