# Supplementary material for: 8-Oxoguanine Affects DNA Backbone Conformation in the EcoRI Recognition Site and Inhibits Its Cleavage by the Enzyme
Source: PLoS One. 2016 Oct 17;11(10):e0164424. doi: 10.1371/journal.pone.0164424 (PMC5066940; doi:10.1371/journal.pone.0164424)
Supplement: S1 Document — (PDF) [file pone.0164424.s001.pdf]

Supplementary Document 1 (S1 Document)

Energy minimization, restraints and simulated annealing protocols for rMD

The starting structures for each sample were subjected to an initial energy minimization of 100 cycles starting with 50 steps using the steepest descent algorithm.

A force constant of 32 kcal mol^{-1} A^{-2} was applied to all NOE and H-bonding restraints. For backbone torsion restraints, a force constant of 32 kcal mol^{-1} deg^{-2} was applied for all angles with the exception of epsilon and zeta of each oxoG nucleotide, which had a force constant of 512 kcal mol^{-1} deg^{-2}.

For rMD, the temperature of the system was increased from 0 K to the high ("target") temperature during the first 5 ps with a coupling periodicity of 0.4 ps. The weight of all restraints was gradually increased from 0.1 to 1.0 over the first 3 ps. The simulated annealing protocols was performed using varying high "target" temperature values (580 K, 600 K and 620 K) and held at that temperature for varying times (90 ps, 100 ps and 110 ps). Cooling the system from the target temperature to 100 K was accomplished over 100 ps with a coupling of 4 ps. The system was cooled from 100 K to 0 K over 10 ps with a coupling of 1.0 to 0.05 ps. The resulting structures were subjected to energy minimization until convergence.

Initialization, minimization and equilibration protocol for fMD simulations

Each system was neutralized with 22 Na^+ ions using CPPTRAJ from AmberTools 13. Additional Na^+ and Cl^- ions were added for a final excess salt concentration of approximately 150 mM. Initial placement of excess ions was randomized using CPPTRAJ by swapping water and ion positions such that no ion was closer than 4 Å to another ion and that all ions were at least 6 Å away from the DNA duplex. Each system followed the same minimization and equilibration protocol. Initially, the DNA atoms were held fixed. To eliminate Van der Waals clashes, the solvent molecules and ions were subjected to 500 steps of the steepest descent minimization. Subsequently, 500 steps of conjugate gradient minimization, with a force constant of 500 kcal/mol-Å^2 applied to the solute molecule. Next, the whole system was minimized with 1000 steps of the steepest descent followed by 1500 steps of conjugate gradient minimization without restraints. Heating was completed over 20 ps at constant volume from 0K to 300K with weak positional restraints (10 kcal/mol-Å^2) applied to the DNA molecule. A 2 fs time step was used and Langevin dynamics (http://dx.doi.org/10.1063/1.1332996) were used to control temperature, with a collision frequency of 1.0 ps^{-1}. SHAKE (doi:10.1016/0021-9991(77)90098-5) was used to constrain bonds involving hydrogen atoms. A 10 Å cutoff was used for non-bonded interactions and Particle Mesh Ewald (PME) was used to handle long range electrostatics. Finally, 100 ps of MD was run at 300K with no restraints and constant pressure to relax the density of water. All other parameters were retained from the previous equilibration.
