# Supplementary figures and images for: 8-Oxoguanine Affects DNA Backbone Conformation in the EcoRI Recognition Site and Inhibits Its Cleavage by the Enzyme
Source: PLoS One. 2016 Oct 17;11(10):e0164424. doi: 10.1371/journal.pone.0164424 (PMC5066940; doi:10.1371/journal.pone.0164424)

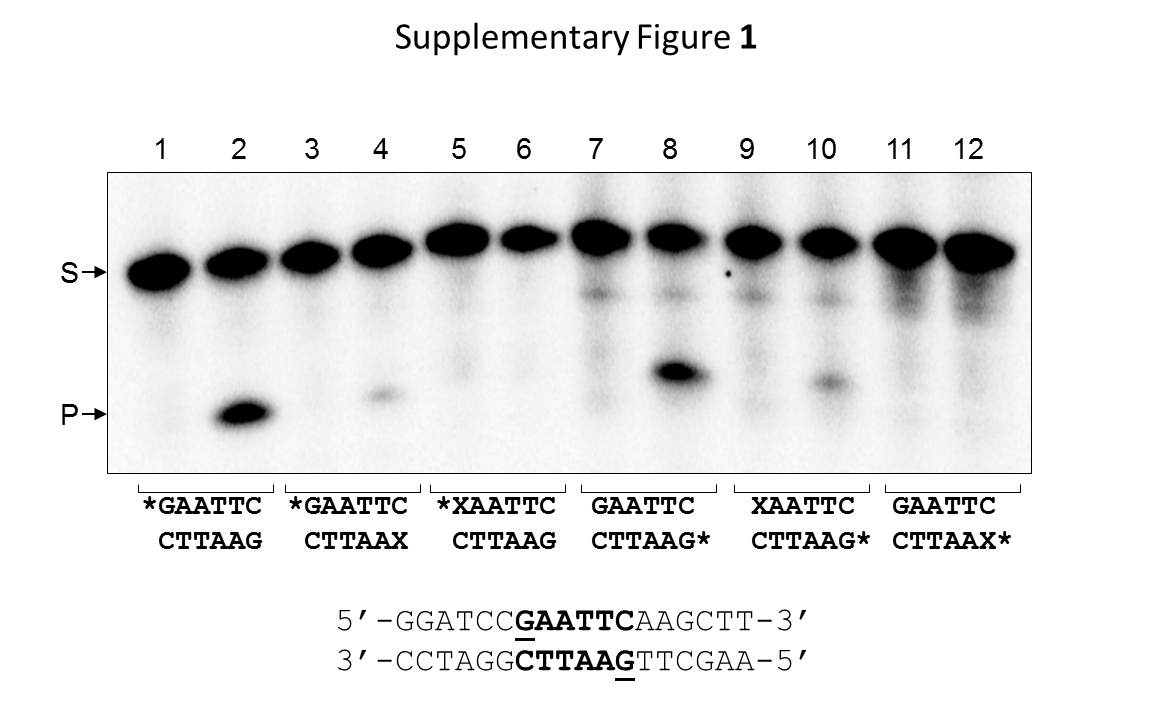

Supplement: S1 Fig — Odd lanes, no enzyme; even lanes, EcoRI. The asterisk marks the 32P-labeled chain. X = oxoG. The sequence of the EcoRI site-bearing duplex is shown. (TIF) [file pone.0164424.s002.tif]

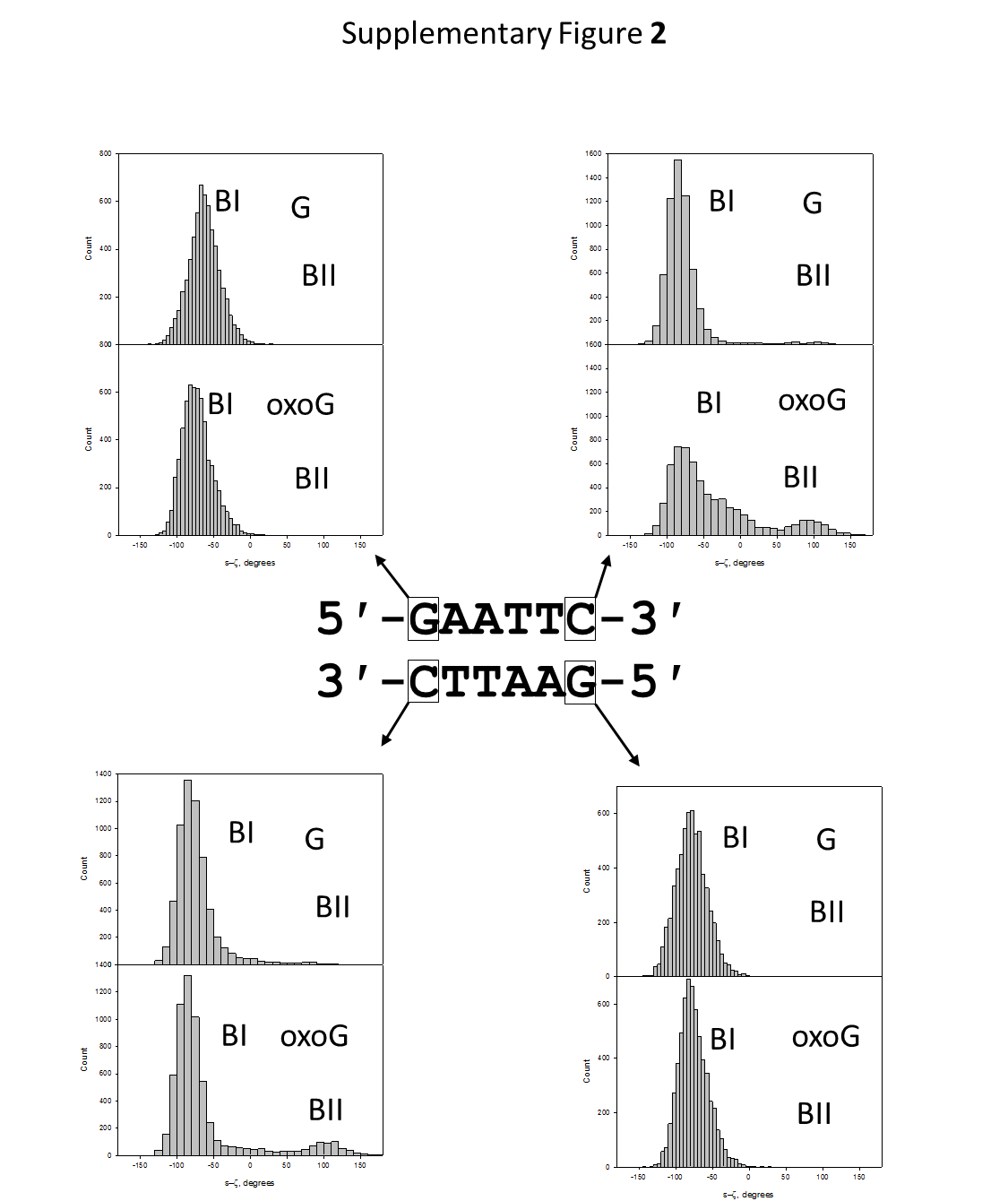

Supplement: S2 Fig — (TIF) [file pone.0164424.s003.tif]

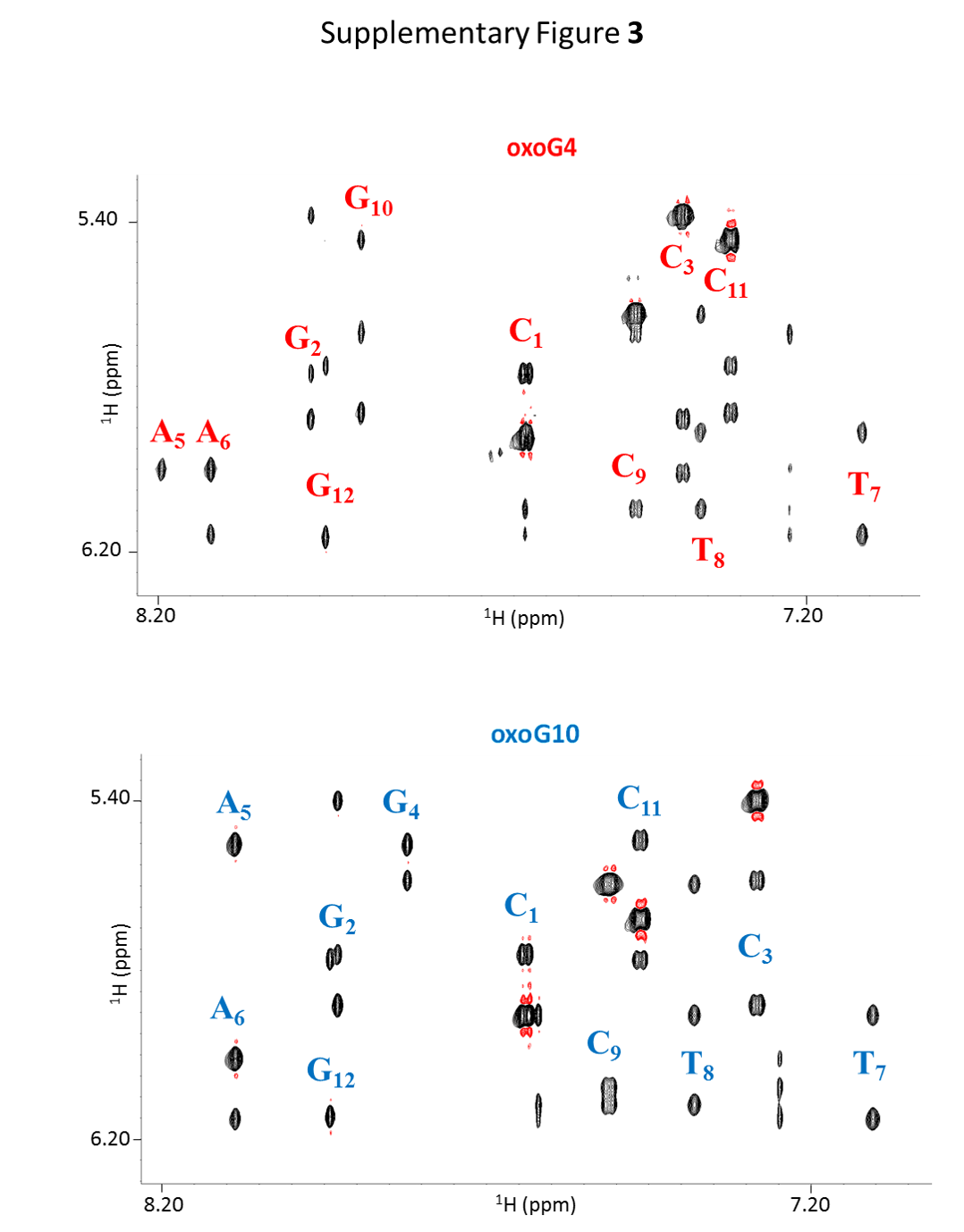

Supplement: S3 Fig — The dodecamer sequence was confirmed to be symmetric, right handed helix within this region. The cross-peaks between the base proton H6/H8 and the H1’ of current or next bases (the “walk”) lead to the NMR resonance assignment (base protons labeled). The number of peaks shown confirms the loss of the guanine H8 at the appropriate modification site for both oxoG4 and oxoG10. (TIF) [file pone.0164424.s004.tif]

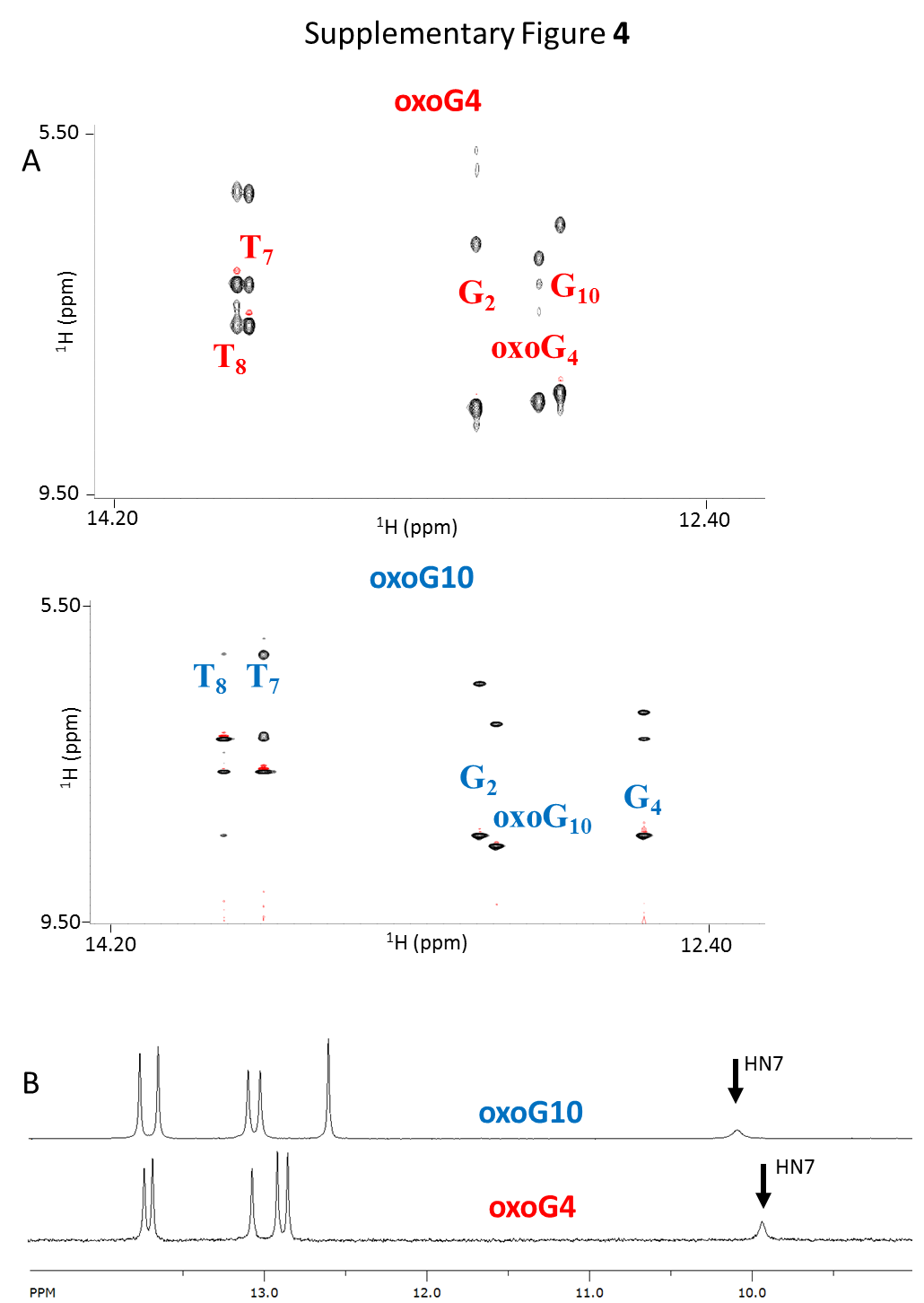

Supplement: S4 Fig — (A) 2D NOESY spectra highlighting the Watson-Crick base pairing interactions in oxoG4 and oxoG10. Three separate CG base pairs are observed for both samples, with the resonances from terminal base pairs not showing due to their decreased thermal stability. Two distinct AT base pairs are also observed for both samples. (B) The imino proton regions of the 1D spectra. The modified residues contain an additional hydrogen at N7 (HN7) that is not seen in canonical guanines. The peaks marked with arrows correspond to the HN7 in both samples. (TIF) [file pone.0164424.s005.tif]

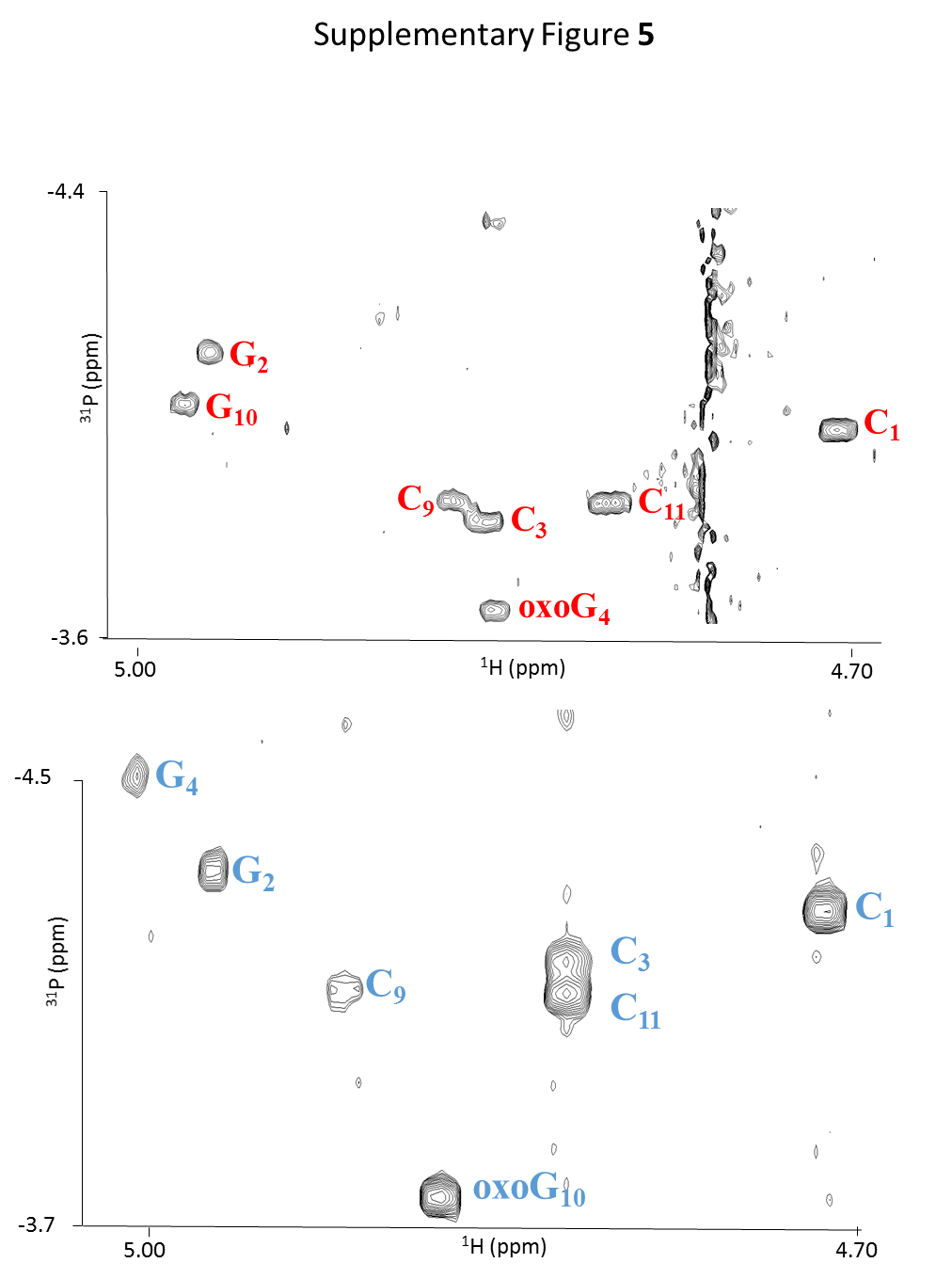

Supplement: S5 Fig — Labels are provided to highlight the residue whose H3' corresponds to that specific peak, all phosphates belong to the residue on the 3' end of the labelled residue. In both spectra, a significant shift is seen in the 31P dimension of the modified residue, demonstrating evidence for that the backbone on the 3' end of the oxidized guanine residue is in BII, and all others are in the canonical BI form. (TIF) [file pone.0164424.s006.tif]

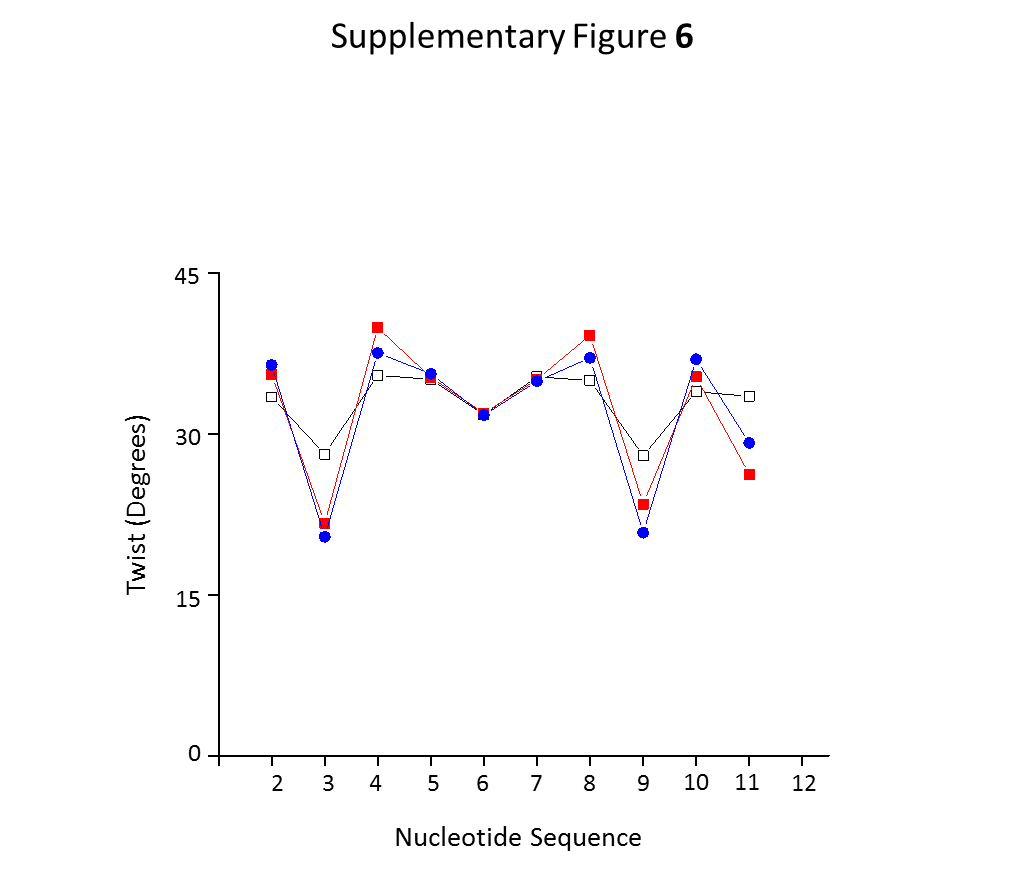

Supplement: S6 Fig — The nucleotide sequence numbers correspond to the steps in the sequence, with the twist for nucleotide step 1 corresponding to the twist of the first CG base pair with respect to the next one. In black with open square markers, are the DDD twist for the unmodified DDD. In red with closed square markers are the twist for oxoG4 and blue with closed circle markers are oxoG10. (TIF) [file pone.0164424.s007.tif]

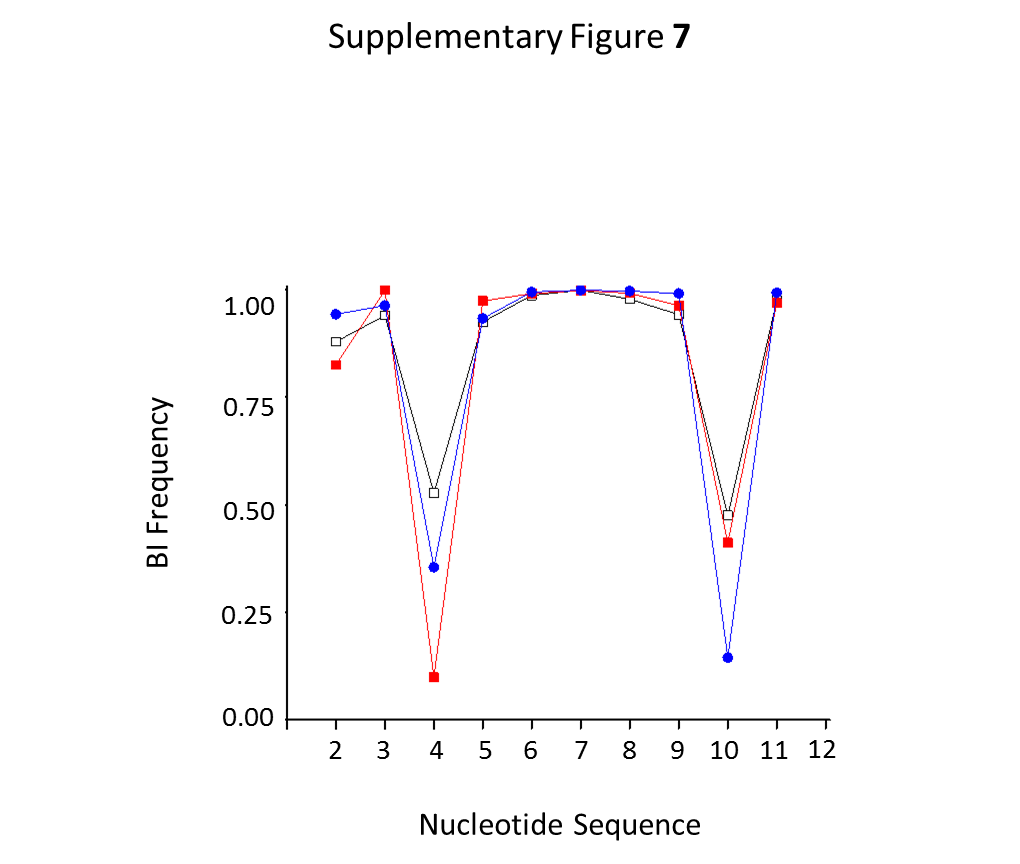

Supplement: S7 Fig — The nucleotide sequence numbers correspond to the steps in the sequence, with the twist for nucleotide step 1 corresponding to the backbone step between the C1 and G2 in DDD sequence. In black with open square markers, are the DDD twist for the unmodified DDD. In red with closed square markers are the twist for oxoG4 and blue with closed circle markers are oxoG10. (TIF) [file pone.0164424.s008.tif]
