# Supplementary material for: 8-Oxoguanine Affects DNA Backbone Conformation in the EcoRI Recognition Site and Inhibits Its Cleavage by the Enzyme
Source: PLoS One. 2016 Oct 17;11(10):e0164424. doi: 10.1371/journal.pone.0164424 (PMC5066940; doi:10.1371/journal.pone.0164424)
Supplement: S1 Table — (PDF) [file pone.0164424.s009.pdf]

S1 Table. Tolerances applied to NOE distance restraints in structure calculations.

Distance Category	oxoG4 Tolerance (Å)	oxoG10 Tolerance (Å)
Short (< 3 Å)	0.50	0.45
Medium (3 Å < x < 5 Å)	0.70	0.65
Long (> 5 Å)	0.90	0.85
Methyl-Containing	1.8 Å	1.8 Å
