# Supplementary material for: 8-Oxoguanine Affects DNA Backbone Conformation in the EcoRI Recognition Site and Inhibits Its Cleavage by the Enzyme
Source: PLoS One. 2016 Oct 17;11(10):e0164424. doi: 10.1371/journal.pone.0164424 (PMC5066940; doi:10.1371/journal.pone.0164424)
Supplement: S2 Table — (PDF) [file pone.0164424.s010.pdf]

S2 Table. Hydrogen bonds observed in the GAATTC site during molecular dynamics simulation of *Eco*RI in a complex with DNA containing G or oxoG at the 1st position of the recognition sequence[*]

Donor	Acceptor	G			oxoG		
		Mean length, Å	Mean energy, kcal/mol	Occurrence, %	Mean length, Å	Mean energy, kcal/mol	Occurrence, %
Protein–DNA bonds							
N[Lys73]	O1P[G/oxoG1]	3.02<br>3.01	–2.26<br>–2.36	99<br>99	2.98<br>2.95	–2.36<br>–2.35	99<br>99
Nζ[Lys97]	O1P[A2]	3.07<br>3.04	–2.27<br>–2.30	96<br>64	3.01<br>3.06	–2.35<br>–2.31	99<br>98
N[His98]	O1P[A3]	3.04<br>3.06	–2.31<br>–2.28	92<br>82	3.09<br>3.08	–2.23<br>–2.23	86<br>78
N[Gly100]	O2P[T4]	3.11<br>3.08	–2.27<br>–2.25	99<br>98	3.09<br>3.07	–2.29<br>–2.29	99<br>99
N[Asn125]	O^4[T4]	3.13<br>3.07	–2.20<br>–2.24	99<br>99	3.10<br>3.08	–2.24<br>–2.24	99<br>99
Nδ2[Asn125]	N7[A2]	3.15<br>3.15	–1.69<br>–1.66	73<br>85	3.14<br>3.12	–1.72<br>–1.80	81<br>97
N[Ala126]	O^4[T4]	3.05<br>3.05	–2.15<br>–2.12	96<br>90	3.06<br>3.06	–2.15<br>–2.14	96<br>94
Nη2[Arg129]	O2P[A2]	3.07<br>3.08	–1.94<br>–2.18	2<br>85	3.07<br>3.06	–2.12<br>–2.29	62<br>98
Nζ[Lys132]	O2P[G/oxoG1]	3.05<br>3.03	–2.27<br>–2.16	93<br>7	3.14<br>3.05	–2.14<br>–2.20	44<br>80
Nη1[Arg187]	O^8[oxoG1]	n/a[#]	n/a	n/a	2.99<br>3.06	–2.33<br>–2.29	99<br>99
N^6[A2]	Oδ1[Asn125]	2.95<br>3.00	–2.36<br>–2.33	99<br>99	3.01<br>3.03	–2.34<br>–2.31	99<br>99
N^4[C6]	O[Ala122]	3.02<br>3.04	–2.29<br>–2.34	99<br>99	3.04<br>3.04	–2.30<br>–2.39	99<br>99
Watson–Crick bonds							
N1[G/oxoG1]	N3[C(6)]	3.11<br>3.05	–1.92<br>–1.91	99<br>99	3.06<br>3.05	–1.87<br>–1.86	98<br>98
N^2[G/oxoG1]	O^2[C(6)]	2.98<br>2.99	–2.33<br>–2.39	99<br>100	3.02<br>2.98	–2.38<br>–2.39	99<br>100
N^6[A2]	O^4[T(5)]	2.93<br>2.95	–2.40<br>–2.39	99<br>99	2.95<br>2.97	–2.40<br>–2.39	99<br>99
N^6[A3]	O^4[T(4)]	3.07<br>3.12	–2.31<br>–2.23	99<br>99	3.06<br>3.07	–2.30<br>–2.27	99<br>99
N3[T4]	N1[A(3)]	3.12<br>3.08	–1.87<br>–1.88	99<br>99	3.17<br>3.13	–1.82<br>–1.85	99<br>99
N3[T5]	N1[A(2)]	3.13<br>3.14	–1.87<br>–1.86	99<br>99	3.12<br>3.14	–1.86<br>–1.85	99<br>99
N^4[C6]	O^6[G/oxoG(1)]	3.05<br>2.95	–2.35<br>–2.40	99<br>100	2.96<br>2.97	–2.39<br>–2.38	99<br>99

[*]The bonds existing in at least one of two half-sites for at least 90% of the simulation are listed. The values for both half-sites are presented. Indexes in parenthesis refer to the complementary bases.

[#]n/a, not applied
